# Identification of immune-relevant candidate genes in atherosclerosis by WGCNA and single-cell analysis

**DOI:** 10.1097/MD.0000000000045871

**Published:** 2025-11-14

**Authors:** Yang Cao, Ying Wei, Rong Xue, Youdong Yang, Weiqin Sun, Wenyuan Dong

**Affiliations:** a Vasculocardiology Department, The Third People’s Hospital of Datong, Datong, China.

**Keywords:** atherosclerosis, immune-related genes, machine learning, single-cell RNA sequencing, WGCNA

## Abstract

Atherosclerosis (AS) is a systemic disease closely related to inflammatory cell infiltration and immune cell activation, often leading to myocardial infarction and stroke and is the leading cause of death worldwide. AS is asymptomatic in its early stages, which leads to a low rate of early diagnosis of the disease and often delays treatment. Therefore, it is extremely important to explore potential biomarkers and molecular mechanisms for the diagnosis and treatment of AS, not only to improve early diagnosis and early treatment of patients but also to reduce the risk of death. The datasets GSE43292 and GSE100927 containing atherosclerotic plaques and normal arterial tissues (including 101 cases of atherosclerotic samples and 66 cases of normal tissue samples) were downloaded from the Gene Expression Omnibus database. The relationship between gene expression and immune cells was analyzed by the CIBERSORT package. Then the differentially expressed genes, weighted gene co-expression network analysis, and immune-related genes (IRGs) set were used to screen out the differentially expressed IRGs. These differentially expressed IRGs were then analyzed by constructing random forest model, support vector machine model, and generalized linear model. Next, a nomogram was established to assess disease risk, the calibration curve, decision curve analysis curve, and clinical impact curve were used to assess the validity of these models. The molecular mechanisms of these biomarkers were analyzed using single-gene gene set enrichment analysis. Potential target drugs for these molecules were identified in the Drug–Gene Interaction database. We screened 5 potential immune-relevant biomarkers (SYK, PTPRC, ITGAL, FGR, and IL10RA) associated with AS, constructed diagnostic models, and predicted potential therapeutic agents. Our findings, derived from integrated bioinformatics analysis, provide novel candidate genes and insights for the future diagnosis and treatment of AS, which warrant further experimental validation.

## 1. Introduction

Atherosclerotic plaques are formed when fatty or fibrous material is deposited in the intima of arteries and gradually calcifies. Advanced atherosclerotic plaque can invade the vessel’s lumen and block blood flow,^[1]^ or atherosclerotic plaques can detach and form blood clots, both of which can cause tissue ischemia and lead to serious consequences. In advanced stages, atherosclerotic plaque can invade the lumen of blood vessels and obstruct blood flow, or atherosclerotic plaque can detach and form a blood clot, both of which can cause tissue ischemia and lead to serious consequences.^[2]^ Atherosclerosis (AS) causes many ischemic strokes and transient ischemic attacks as well as some peripheral vascular diseases, and the atherosclerotic cardiovascular disease has the highest incidence, which can lead to acute coronary syndromes and endanger patients’ lives. AS remains a major fatal disease worldwide, because AS is asymptomatic in its early stages, patients are often diagnosed with advanced AS when plaques block blood vessels causing ischemic symptoms, or when plaques dislodge and embolize blood vessels causing tissue hypoxia.^[3]^ Although some invasive diagnostic tests (e.g. coronary angiography) and noninvasive tests (e.g. ultrasound) are available, rapid, and noninvasive biological markers are still rarely used in clinical practice.^[4]^ Therefore, it is an urgent clinical issue to study the underlying mechanisms of AS and search for effective noninvasive biomarkers. The pathogenesis of AS is not fully understood. AS is generally considered to be a disease of cholesterol accumulation caused by the deposition of lipoproteins, including low-density lipoproteins (LDL), in the endothelium of blood vessels.^[5]^ The absorption of LDL by scavenger receptor causes a continuous infiltration of immune cells into the atherosclerotic plaque, in which macrophages engulf a large number of lipoproteins and then become foam cells, the process of AS is accompanied by the close involvement of immune cells. Genome-wide associations, clonal lineage tracking, and clinical trial analyses suggest that innate and adaptive immunity can promote or inhibit AS.^[6]^ Recently, single-cell transcriptomics and proteomics analyses identified immune cell dysregulation in atherosclerotic plaques.^[7]^ CD4 + T cell subsets and T cells were activated in the plaques of symptomatic patients, while T cells and macrophages were activated in the plaques of asymptomatic patients. All of these studies demonstrate the functional and phenotypic diversity of immune cells in atherosclerotic plaques, as well as the interaction of systemic immune responses and local plaques that cause AS.^[8]^ Targeted treatment of AS by specifying the dysregulation of specific immunity within the plaque region is becoming a new therapeutic trend. Also, combining differential analysis of immune cell-related genes is a new idea to diagnose and treat the disease, in this study, we from the Gene Expression Omnibus database (GEO, http://www.ncbi.nlm.nih.gov/geo/) to download the data sets of AS (GSE43292 and GSE100927), to explore the potential of biomarkers. We aimed to identify candidate biomarkers, construct diagnostic models, and predict potential therapeutic agents associated with AS by using bioinformatics analysis.

## 2. Materials and methods

### 2.1. Data source

From the GEO database (https://www.ncbi.nlm.nih.gov/geo/) download GSE43292 and GSE100927 data sets, extracting Gene Expression matrix. The GSE43292 dataset contains transcriptome sequencing data from 32 atherosclerotic and 32 normal vascular tissue samples. The GSE100927 dataset includes transcriptome sequencing data from 69 atherosclerotic and 34 normal vascular tissue samples. We get from import database (https://www.immport.org/home), immune-related genes (IRGs).^[9]^

### 2.2. Data preprocessing

First merge the 2 data sets and then use the “sva” R package (Bioconductor, Seattle) to remove the batch effect.^[10]^ The expression matrices from different batches or platforms were then normalized by the “ComBat” method to remove the batch effects from both datasets.

### 2.3. Immune cell infiltration analysis

CIBERSORT algorithm and immune cell LM22 gene set were used to calculate the relative abundance of 22 immune cells in AS and normal samples.^[11]^ After excluding samples with no statistical significance (*P*-value > .0001), the 22 immune cells were compared by the Wilcoxon test, and the Wilcoxon test was used for comparison.

### 2.4. Weighted gene co-expression network analysis

We used the weighted gene co-expression network analysis (“WGCNA”) package of R software^[12]^ to analyze expression data in normal and atherosclerotic samples. The “goodSamplesGenes” function is used to cluster samples to identify and remove outliers. To make the co-expression network satisfy the distribution of a scale-free network, soft threshold power is calculated by selecting the soft threshold function. The dynamic tree-cutting method was used to identify different modules. The minimum gene number of each module was set to 30, and the modules were screened. We then combined these modules and further analyzed the correlation between the combined final modules and differential immune cells. The modules with the highest correlation with differential immune cells were extracted (|correlation coefficient |>0.5, *P*-value < .05), and the genes satisfying |MM|>0.8 and |GS|>0.4 were taken as key module genes. MM represents the correlation between genes and modules in a module, and GS represents the correlation between genes and traits.

### 2.5. Identification and functional enrichment analysis of differentially expressed genes (DEGs)

Differential genes between normal and atherosclerotic samples were filtered using the “limma” packet.^[13]^ The threshold value is |log2FoldChange| > 1 and the adjusted *P*-value < for .05. Heat maps of differential genes were created using the ComplexHeatmap package. Using the “clusterProfiler” package to perform the Kyoto Encyclopedia of Genes and Genomes (KEGG) pathway enrichment analysis.^[14]^ Pathways with adjusted *P*-value < .05 in the enrichment analysis were considered to be by significance.

### 2.6. Screening biomarkers by machine learning models

First, we obtained the key modules of WGCNA, then intersected the key templates to obtain the differentially expressed immune cell-related genes, and then took the intersection of module intersection genes, DEGs, and IRGs. Based on differentially 2/10 expressed immune cell-related genes, 3 machine learning models, random forest model (RF), support vector machine model, and generalized linear model, were constructed using caret R package. Screened differentially expressed immune cell-associated genes were used as explanatory variables and AS samples were used as response variables.^[15]^ The “explain” function in the “DALEX” R package is used to analyze the 3 models, and the cumulative residual distribution is plotted to obtain the best model. Finally, we analyzed the importance of these differentially expressed immune cell-related genes in predicting the response variable (AS).

### 2.7. Construction and validation of diagnostic map of AS

After selecting differentially expressed immune cell-related genes using RF models, Cytoscape software was used to screen hub genes in these DEGs, and then a histogram was drawn according to the selected hub genes for clinical assessment of AS development. Calibration curves were then drawn to evaluate the predictive accuracy of the column line graphs. Moreover, the decision curve and clinical impact curve were drawn to evaluate the clinical value of the histogram.

### 2.8. Single gene set enrichment analysis (GSEA)

To explore the potential function of the biomarkers,^[16]^ we performed a single GSEA of the screened differential genes. Gene Ontology (GO) enrichment analysis mainly describes gene-related biological processes (BP), cellular components (CC), and molecular functions (MF). According to the expression of each gene, the correlation coefficient of each gene with all the genes in the gene set is sequenced. The enrichment significance threshold was NOM < 0.05.

### 2.9. General analyses of single-cell sequencing data

AS dataset GSE159677 single-cell transcriptome data was obtained from the GEO database. The following genes were excluded: genes expressed in fewer than 10 cells were excluded from subsequent analyses. The following cells were excluded: percentage of mitochondrial genes >5%; number of characterized genes <200; and number of reads <2000. The 13 principal components were visualized using uniform manifold approximation and projection plot. Annotation of cell types using SingleR.

### 2.10. Prediction of potential drugs

Drug–Gene Interaction Database (DGIdb) (https://www.dgidb.org/search) to analyze the differences of the screening of genes, to predict potential drugs for the treatment of AS. The DGIdb database (https://www.dgidb.org/search interactions) was used to analyze the differential genes screened to predict potential drugs for the treatment of AS.^[17]^ The network structure of biomarker–compound pairs was visualized using the networkD3 package.

## 3. Results

### 3.1. Immune cell infiltration between atherosclerotic and normal samples

**Figure 1.**
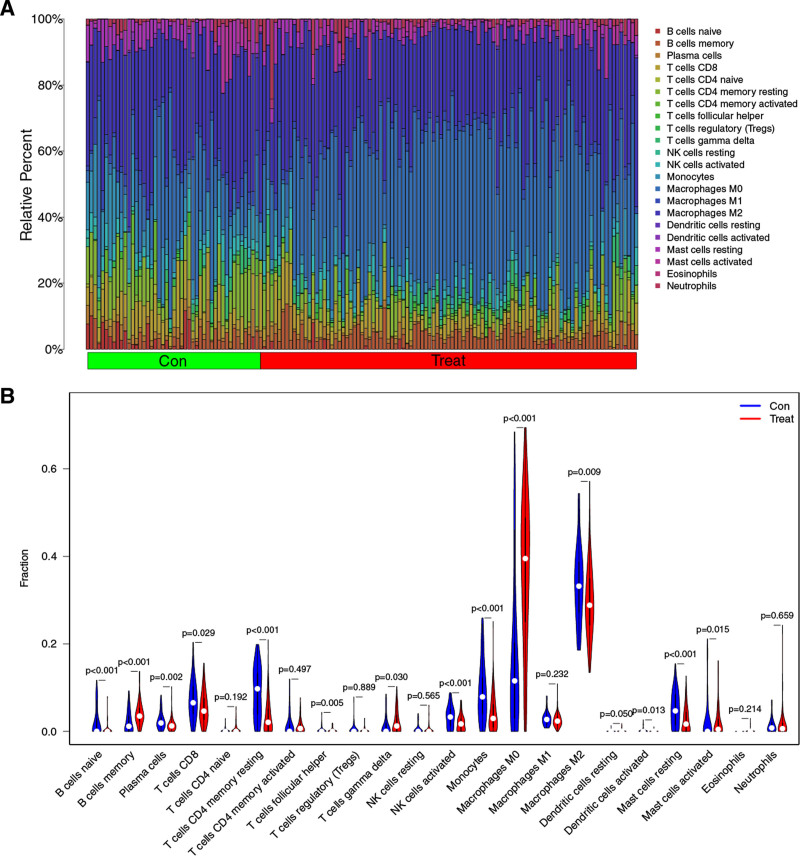
The immune infiltration cell analysis between atherosclerosis (AS) and control samples (Con). (A) Proportion of atherosclerosis and control samples. The different colors represent different immune cells and the size of the pillar represents the proportion of immune cells. (B) Differences between AS and Con samples for 22 infiltration immune cells. Red represents atherosclerosis (AS), blue represents control samples (Con).

Two AS datasets (GSE43292 and GSE100927) were selected for the study, and we used Combat method to eliminate the batch effect between the 2 datasets. We finally obtained normalized expression data for 101 AS and 66 normal samples (NC). We then used the CIBERSORT algorithm to calculate the proportion of 22 immune cell infiltrates in each sample. Twenty samples with insignificant results were eliminated, and only 147 samples with *P*-values < .05 were retained. Figure 1A shows the percentage of immune cell infiltration in AS and NC samples. As shown in Figure 1B, macrophage M0 and memory B cells in the AS group were significantly higher than those in the NC group, while macrophages M2, CD4 + T cells memory resting, B cells naive, Mast cells resting, natural killer (NK) cells activated and monocytes were significantly lower than those in the NC group. The results showed that there were significant differences in the immune microenvironment between the AS group and the NC group.

### 3.2. Discovery of differentially expressed immune cell-associated genes using WGCNA

**Figure 2.**
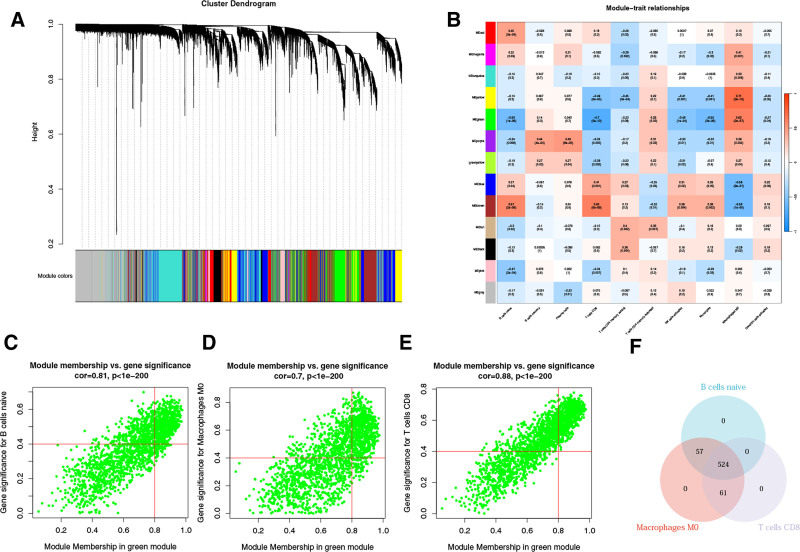
Weighted gene co-expression network analysis based on differential immune infiltration cells. (A) Cluster dendrogram. Genes are divided into various modules by hierarchical clustering, and different colors represent different modules, among which gray defaults to genes that cannot be classified into any module. (B) The heatmap of modules and immune cells correlation. In the figure, red represents positive correlation, blue represents negative correlation, the darker the color, the stronger the correlation. Each frame labels the correlation coefficient, with the corresponding *P*-value in brackets. (C–E) The scatter plots to show the correlation for MM (X-axis) and GS (Y-axis) in B cells naive (C), macrophages M0 (D), and T cells CD8 (E) for the genes in the green module. (F) Venn diagrams. Five hundred twenty-four genes (intersection genes) related to all 3 types of immune cells were obtained by taking the intersection.

To search for genes associated with differential immune cells, we performed WGCNA on genes from 22 differential immune cells from 100 AS samples and 47 NC samples. Samples were not removed at the time of sample clustering. Next, select the soft threshold power β = 13 (scale-free *R*^2^ = 0.95) to construct the scale-free network (Figure S1, Supplemental Digital Content, https://links.lww.com/MD/Q630). Then, a cluster dendrogram was constructed and a dynamic tree-cutting was performed (Fig. 2A). Subsequently, after merging the final 7 modules and assessing the correlation between each module and differential immune cells, the immune cells with the highest correlation with the modules were screened based on the correlation (Fig. 2B). According to the standard |cor| > 0.5 and *P*-value < .05, it was analyzed that turquoise modules and B cells naive, T cells CD4 memory resting, and macrophages M0 were significantly correlated, and among the key modules, expressed genes with |MM| > 0.8 and |GS| > 0.4 were considered as key module genes. A total of 1009 macrophages of M0-related key module genes, 706 B cells of naive-related key module genes, and 786 T cells of CD4 memory resting-related key module genes were screened (Fig. 2C–E). Finally, by taking 3/10 the intersection of the 3 groups of genes, we obtained 599 IRGs that are differentially expressed in AS (Fig. 2F).

### 3.3. Identification of diagnostic genes

**Figure 3.**
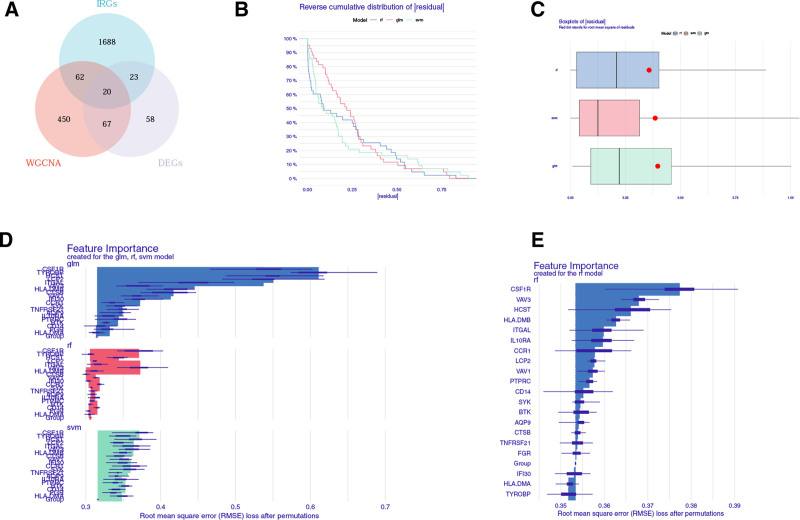
Establishment of diagnostic models and identification of potential diagnostic biomarkers. (A) Venn diagrams for differentially expressed immune cell-related genes. (B) Cumulative residual distribution plot of sample. (C) Boxplot of the residuals of the sample. (D–E) The importance of variables in GLM, RF and SVM models. GLM = generalized linear model, RF = random forest, SVM = support vector machine.

We screened DEGs between AS and NC samples using the “limma” software package and showed that 168 DEGs were identified in AS, including 124 up-regulated genes and 44 down-regulated genes. In order to obtain the DEGs associated with immune cells in AS, we took the intersection of IRGs, differential immune cell-associated genes, and DEGs (Fig. 3A), obtained 20 differential genes, and identified biomarkers by using 3 machine learning analysis methods, namely: random forest (RF), support vector machine model, and generalized linear model, which created independent models based on 100 AS samples and 47 NC samples, respectively. Then, the “DALEX” package with explanatory features was used to analyze the above 3 models, and the residual distribution based on 100 AS samples and 47 NC samples was drawn to obtain the optimal model. Based on the cumulative residual distributions and box plots, RF had the smallest sample residuals and was the best model (Fig. 3B, C). We then analyzed the importance of these variables (20 genes) in predicting response variables (AS or NC) and selected the 17 most important explanatory variables from the RF model for further analysis (Fig. 3D, E). Analysis of 17 genes using Cytoscape software yielded the 5 most important hub genes as potential biomarkers for the disease (Figure S2, Supplemental Digital Content, https://links.lww.com/MD/Q630).

### 3.4. Construction and evaluation of a columnar map for the diagnosis of endometriosis

**Figure 4.**
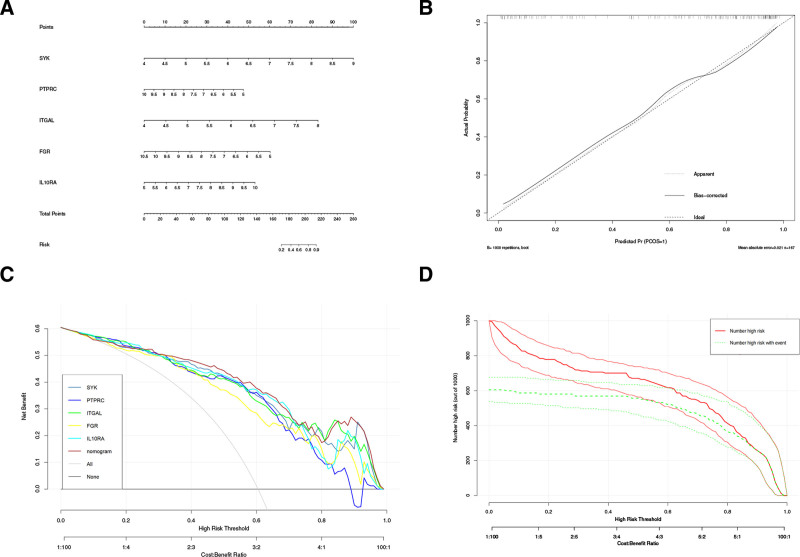
Construction and validation of a nomogram model for EM diagnosis. (A) The nomogram of diagnostic biomarkers to predict the occurrence of EM. (B) The calibration curve to assess the predictive power of the nomogram model. (C) The DCA curve to evaluate the clinical application value of the nomogram model. (D) Clinical impact curves of the nomogram model. DCA = decision curve analysis.

Next, we used the “RMS” package to create a column line plot for predicting AS based on 5 biomarkers (Fig. 4A). The correction curve showed a small error between the actual risk of AS and the predicted risk, while the histogram showed a high prediction accuracy (Fig. 4B). “PTPRC” curve, “ITGAL” curve, “FGR” curve, and “IL10RA” curves, indicating that between 0.7 and 1.0, patients could benefit from a columnar plot with a high risk threshold (Fig. 4C). On the basis of the decision curve analysis curve, we further draw the clinical impact curve of the nomogram model, so that the clinical effect of the histogram can be evaluated more intuitively. When the high risk threshold is between 0.4 and 1, the “number high risk” curve is close to the “number high DC risk with event” curve, indicating that nomogram has strong prediction ability (Fig. 4D). These results suggest that these 5 genes may play an important role in the development of AS.

### 3.5. GSEA analysis of single genes

**Figure 5.**
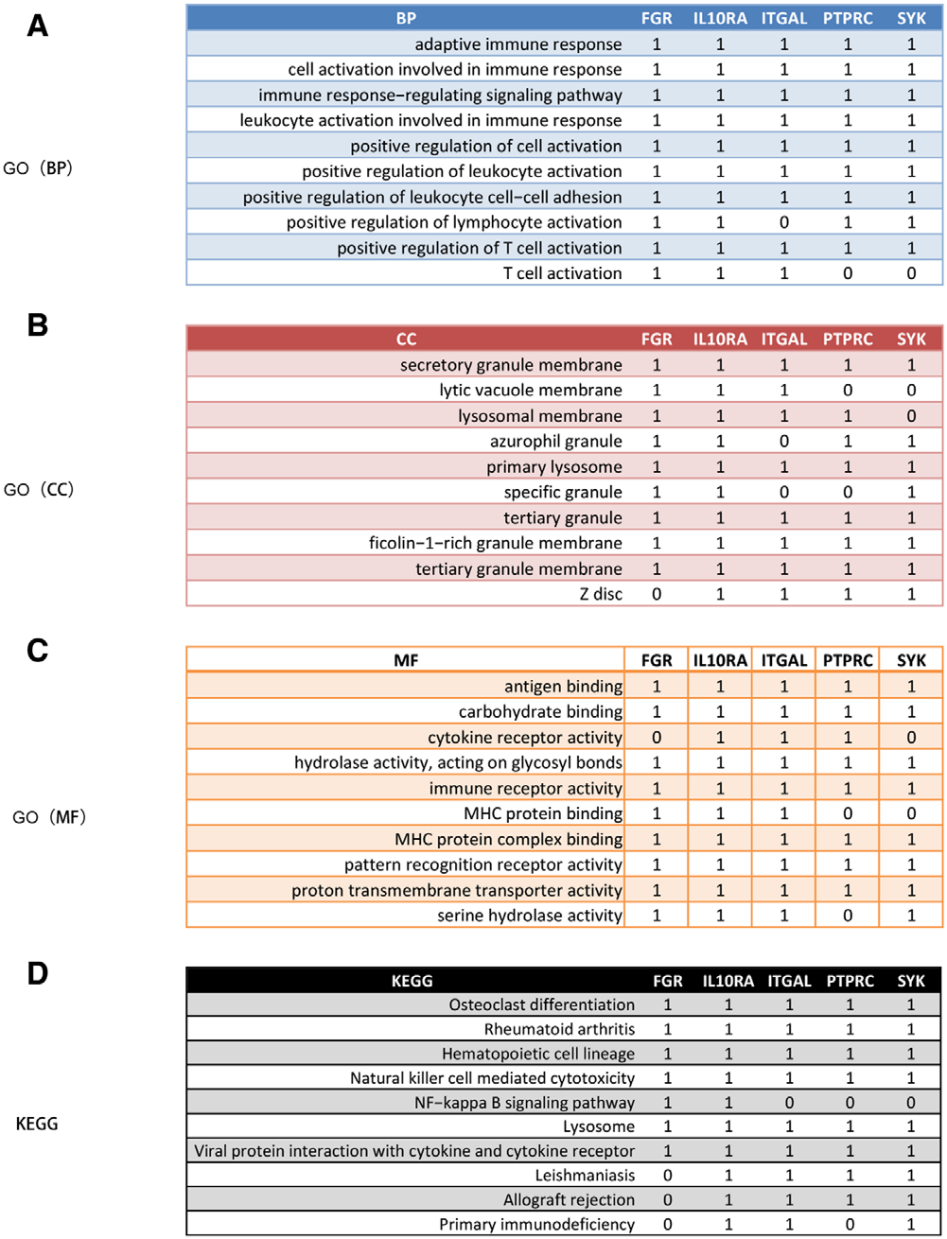
The co-enriched GO entries and KEGG pathways by 5 diagnostic biomarkers via single-gene GSEA. (A–C) Co-enriched GO terms in the B P, CC, and MF categories. (D) Co-enriched KEGG pathways. BP = biological process, CC = cellular component, GO = Gene Ontology, GSEA = gene set enrichment analysis, KEGG = Kyoto Encyclopedia of Genes and Genomes, MF = molecular function, RF = random forest.

Next, we performed GSEA analysis of single biomarkers based on GO and KEGG gene sets to explore the BPs and mechanisms of development during AS, and Figure 5A–C shows the top 10 items associated with all 5 biomarkers in the GO (BP), GO (CC), and GO (MF) categories. In BP, 5 diagnostic genes are collectively involved in adaptive immune response, cell activation involved in immune response, immune response-regulating signaling pathway, leukocyte activation involved in immune response, positive regulation of cell activation, positive regulation of leukocyte activation, positive regulation of leukocyte cell–cell adhesion, and positive regulation of T cell activation. In CC, 5 diagnostic genes are jointly involved in the secretory granule membrane, primary lysosome, tertiary granule, ficolin-1-rich granule membrane, and tertiary granule membrane. In MF, 5 diagnostic genes are jointly involved in antigen binding, carbohydrate binding, hydrolase activity, acting on glycosyl bonds, immune receptor activity, MHC protein complex binding, pattern recognition receptor activity, and proton transmembrane transporter activity. For the KEGG pathway (Fig. 5D), 5 diagnostic genes are collectively involved in osteoclast differentiation, rheumatoid arthritis, tematopoietic cell lineage, NK cell mediated cytotoxicity, lysosome, and viral protein interaction with cytokine and cytokine receptor.

### 3.6. General analysis and annotation of cells at atherosclerotic plaque sites

**Figure 6.**
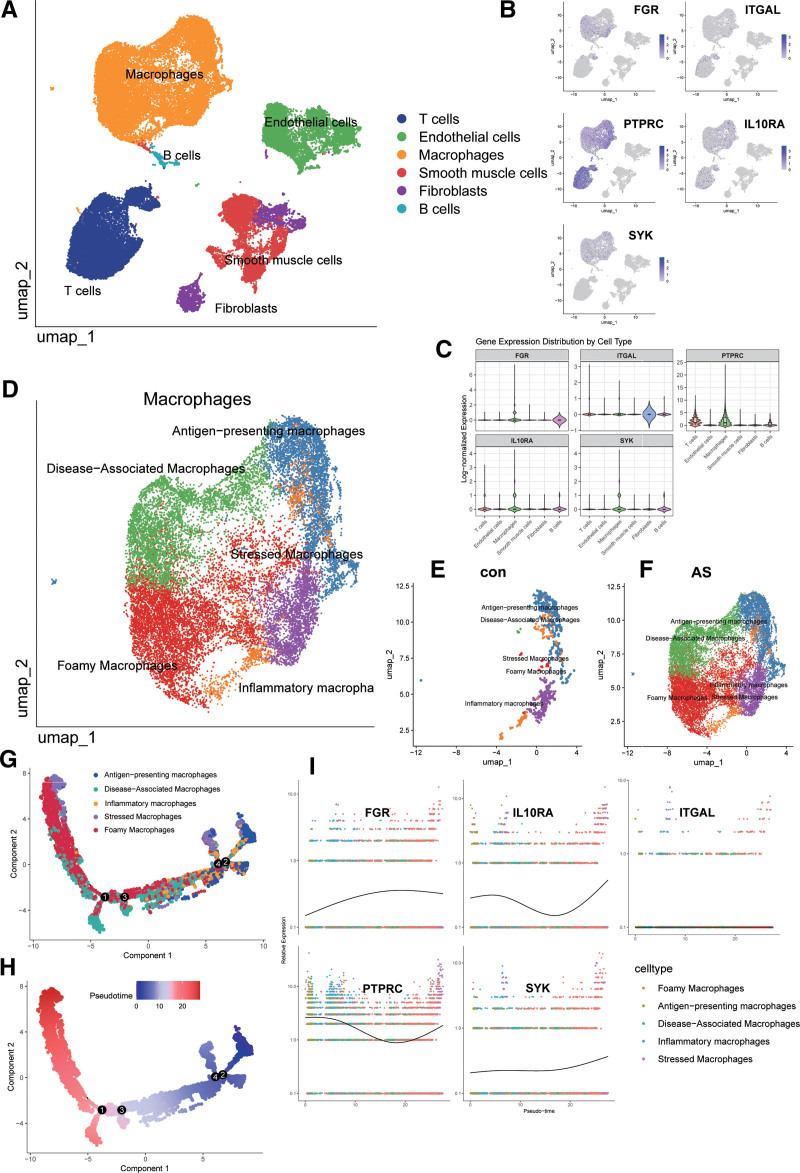
Single-cell analysis of cells at atherosclerotic plaque sites. (A) Cell type annotation using SingleR and marker genes. (B) Expression distribution of screened differentially expressed genes (DEGs) in immune cells. (C) Expression differences of DEGs across cell types. (D) Subtype annotation of macrophages. (E, F) Macrophage subtypes in normal and atherosclerotic (AS) tissues. (G, H) Pseudotime analysis of macrophage subtypes. (I) Expression trends of DEGs along the pseudotime trajectory.

To further investigate immune-related DEGs associated with AS, we performed single-cell RNA sequencing (scRNA-seq) analysis using data from the GSE159677 database, conducting cell clustering and annotation with SingleR (Fig. 6A), and analyzed the expression distribution of the 5 identified DEGs across various cell types (Fig. 6B); FGR, SYK, and IL10RA were predominantly expressed in macrophages and monocytes, PTPRC was highly expressed in macrophages, monocytes, and T cells, while ITGAL exhibited no significant expression in any cell type, and further comparison revealed that these DEGs were most prominently up-regulated in macrophages (Fig. 6C); given the critical role of macrophages in AS pathogenesis, we performed subclustering and functional annotation of macrophage subsets (Fig. 6D), revealing a significant increase in Foamy Macrophages and Disease-Associated Macrophages in AS patients compared to controls (Fig. 6E and F); to explore the dynamic transition of macrophage subtypes, we conducted pseudotime trajectory analysis using Monocle2 (Fig. 6G and H), which indicated a developmental trajectory originating from antigen-presenting macrophages and progressing toward a foamy macrophage phenotype, with all 5 DEGs (except ITGAL) exhibiting dynamic expression patterns along this trajectory, suggesting their potential involvement in macrophage phenotypic transition (Fig. 6I); finally, we validated the diagnostic performance of the 5-gene signature using an independent dataset (GSE132651), and the results demonstrated that the combined expression profile of these genes effectively distinguished AS patients from healthy controls with high accuracy (AUC > 0.85, Figure S3, Supplemental Digital Content, https://links.lww.com/MD/Q630), highlighting its potential for clinical application.

### 3.7. Potential drugs targeting diagnostic genes

To explore potential drugs for the treatment of AS, we searched the DGIdb database for potential drugs targeting these biomarkers. As shown in Table S1, Supplemental Digital Content, https://links.lww.com/MD/Q629, we found that there were 28 drugs targeting SKY, 12 drugs targeting PTPRC, 17 drugs targeting ITGAL, and 6 drugs targeting FGR. Two of these drugs have been used in clinical trials, and these drugs offer potential therapeutic directions for the treatment of AS.

## 4. Discussion

AS is the most common cause of coronary artery disease, cerebrovascular disease, and peripheral artery disease, which often occurs in the intima of medium and large arteries throughout the body. Cerebrovascular disease and peripheral artery disease, which often lead to serious complications such as myocardial infarction, stroke, and intermittent claudication, and is the most common cause of death worldwide.^[18]^ Currently, the global incidence of AS is on a significant rise, but because the early stages of atherosclerotic lesions are basically asymptomatic, early diagnosis of AS is very difficult, and the diagnosis and treatment of AS has been the focus of research and a hot spot in recent years. Typically, AS is a disease of cholesterol storage caused by the deposition of lipoproteins, including LDL, in the intima of arteries, which are absorbed by scavenger receptor-mediated phagocytosis.^[19]^ New research has found that AS is accompanied by a chronic, low-grade inflammatory response that attracts cells from the innate and adaptive immune systems to atherosclerotic plaques, so AS is also an autoimmune chronic inflammatory.^[20]^ Single-cell sequencing shows that human carotid plaques and mouse arterial plaques are composed mainly of macrophages, T cells, and monocytes.^[8]^

In this research, we performed bioinformatic analysis of transcriptomic data from NC and atherosclerotic samples (AS) in the GEO database (GSE43292 and GSE100927 datasets) to identify candidate disease-associated immune genes and explore potential therapeutic agents. First, immunoinfiltration analysis was performed to compare the difference of immune cells in normal and atherosclerotic samples, and a total of 7 significantly different immune cells were identified: macrophages M0, macrophages M2, CD4 + T cells memory resting, B cells naive, Mast cells resting, NK cells activated, and monocytes. Atherogenesis and progression of AS are closely associated with macrophages, oxidized LDL (oxLDL) is an important marker of plaque inflammation, LDL is modified by oxidation of reactive oxygen species to oxLDL and promotes oxLDL into macrophages.^[21]^ Natural LDL can also be absorbed by macrophages as aggregates of cholesterol complexes or crystals through microcellular action or phagocytosis.^[22]^ In addition, native LDL can be absorbed by macrophages through micropinocytosis, and macrophages that phagocytose large amounts of LDL turn into foam cells. In atherosclerotic samples, the content of M0 macrophages increased significantly, and a large number of M0 macrophages turned into foam cells and deposited in the endothelium, leading to the formation of arterial plaques. In contrast, the content of macrophages M2, which has the effect of inhibiting inflammation and promoting tissue repair, was significantly decreased in the arterial plaques. T cells are key regulators of the adaptive immune response and can differentiate into distinct T-helper subtypes, and these subtypes can not only suppress immunity but also activate other T cells, which in turn have direct anti-inflammatory or pro-inflammatory effects on tissue cells. T cells are also found in atherosclerotic plaques, and in AS-prone Apoe-/- cd11c - yfp + mice, stimulated by hypercholesterolemia, antigen-presenting cells interact extensively with CD4 + T cells in the aorta, resulting in cell activation and proliferation, as well as the secretion of IFN-γ and TNF-α, cytokines that promote AS by maintaining chronic inflammation and inducing foam cell formation.^[23]^ T cells are present in all stages of atherosclerotic disease development, and T cell activation and oligoclonal expansion are particularly prominent at plaque rupture sites.^[24]^ B cells can be divided into 2 categories: B1 cells are part of the innate immune system and produce IgM antibodies in a way that does not depend on T cells; B2 cells require activation of T-helper cells to differentiate into plasma cells and produce IgG antibodies. B cells secrete a large number of cytokines that play a significant role in inflammation: immune response activator B can secrete granulocyte-monocyte colony stimulating factor that exacerbates AS.^[25]^ In general, B1 cells seem to have a protective effect against AS and B2 cells have a proatherosclerotic effect. In the present study, we observed a reduce in the abundance of macrophages M2, CD4 + T cells memory resting, and B cells naive in atherosclerotic plaques compared to normal intima, however, the abundance of macrophages M0, T cells activated, and B cells memory significantly increased, which is consistent with previous findings.^[26]^ When stimulated by atherosclerotic lesions, monocytes differentiate into macrophages of different phenotypes, but macrophages in plaques are not exclusively derived from monocytes but mainly rely on their own local proliferation.^[27]^ Most studies suggest that monocytes promote phenotypic transition in vascular smooth muscle cells by activating the NLRP3 inflammasome and that NLRP3 inflammatory vesicles may play a deleterious role in the stability of arterial plaques.^[28]^ Mast cells are migratory cells in connective tissue. There are many particles in mast cells, which are rich in histamine and heparin. Studies have shown that mast cells also play an important role in AS, and mast cells are also present in the intima and epicardial plaques of patients’ aorta.^[29]^ NK cells play a major role in immune regulation in the pathogenesis of AS, and reduced counts and abnormal activity of NK cells have been observed in both coronary artery disease and end-stage renal disease.^[30]^ However, there is no conclusive evidence on the role of mast cells and NK cells in arterial plaque formation.^[31]^

We need a deeper understanding of the role that various immune cells play in the mechanisms of atherosclerotic plaque formation, which will enable the discovery of potential targets and therapeutic directions for AS. Early-stage atheromatous plaques are generally asymptomatic and their pathogenesis has not been fully elucidated. At present, no sensitive and specific biomarkers have been found for the early diagnosis of AS.^[32]^ The gold standard for the diagnosis of AS is CT angiography, but the limitations of technical means often lead to delays in the diagnosis and treatment of the disease, which seriously hinders early detection and treatment. Therefore, it is of great significance to explore noninvasive biomarkers for the clinical diagnosis and treatment of AS. In order to find biomarkers related to the diagnosis of AS, in this study, 3 machine learning methods were used to construct 3 diagnostic models to screen diagnostic genes. The results show that the optimal model of the 3 machine learning algorithms is the RF model: a total of 15 genes with a high impact on the diagnosis of the disease were screened.^[33]^ Five hub genes were then screened by Cytoscape analysis: SYK, PTPRC, ITGAL, FGR, and IL10RA. We established a nomogram for AS using these 5 genes as diagnostic indicators, and verified the nomogram by calibration curve and decision curve, and the results showed good diagnostic accuracy. Syk is a tyrosine kinase that binds the immunoreceptor tyrosine-based activation motif of the FcR-γ chain, which clusters numerous proteins. The activation of Syk leads to the activation of various kinases, such as phosphatidylinositol-3-kinase and phospholipase C-γ2 (PLC-γ2), and release of Ca^2+^ from intracellular stores in activated platelets. Although increased platelet activity increases the risk of AS, pro-inflammatory molecules secreted by platelets play an important role in the development of atherosclerotic inflammation, so inhibition of platelet activation is a potential treatment for AS,^[34]^ PTPRC is one of the most abundantly expressed glycoproteins on the surface of leukocytes and is expressed only in cells of the hematopoietic system. PTPRC has been shown to be an important regulator of T cell and b cell antigen receptor signaling. CD45 has a tendency to be highly expressed in immune cells, including lymphocytes and macrophages, and is a well-known regulator of the inflammatory response, while AS is also considered a chronic inflammatory disease, and inhibition of CD45 expression has potential therapeutic value for AS. ITGAL (CD11a) belongs to the integrin family, which is a receptor of the intercellular adhesion molecule (ICAM) family, it can play a central role in intercellular adhesion of leukocytes through interaction with its ligands ICAMs 1 to 3 and plays a role in lymphocyte co-stimulatory signaling and promotes phagocytosis of apoptotic neutrophils by macrophages together with ICAM3.^[35]^ FGR is a member of the Src family of protein tyrosine kinases. FGR coordinates paracrine signaling in cardiac myocytes and promotes pressure overload-induced angiogenesis,^[36]^ IL10RA is a receptor for interleukin-10. It has been found that it can mediate the immunosuppressive signal of interleukin-10, thus inhibiting the synthesis of pro-inflammatory cytokines. IL10RA may be associated with the pathogenesis of inflammatory diseases.^[37]^ There are few studies on the relationship between these genes and AS, and they may play a role in the pathogenesis of AS, but the exact mechanism needs to be further studied. To further understand the association of these differential genes with immune cells, this study further validated the distribution of differential gene expression in immune cells using a single-cell dataset, which showed that the differential genes were predominantly distributed in macrophages, monocytes, and T cells. The diagnostic efficacy of the 5 differential genes was verified using a validation dataset, and although the diagnostic efficacy of the individual genes was not high, the combined analysis of the 5 genes provided good diagnostic efficacy. Currently, known drug treatments are mainly preventive, preventing the development of AS by lowering high-level factors such as blood lipid levels, or surgical removal of blood vessels at the site of the lesion. There are still no drugs that directly treat atherosclerotic platelets,^[38]^ we screened 5 immune-related biomarkers of AS obtained as potential targets for drug therapy, the DGIdb database was used to predict potential drugs for AS treatment, and visualized a network of diagnostic marker–molecular compound relationship pairs using the networkD3 package. Some of the drugs in the drug network we investigated are already in clinical use, and we labeled them 1 by 1. In addition, it is worth noting that the present study used a combined diagnosis of AS using 5 IRGs, although the price of gene sequencing technology has gradually declined with advances in technology, large-scale clinical genetic testing is still unattainable at present. The aim of this study is to propose a possible idea to help the clinic in disease prediction and diagnosis. Five biomarkers for AS diagnosis still need further research, and large-scale genetic testing still needs technological advances to advance. Several limitations of this study should be acknowledged. Firstly, our analysis is primarily based on bioinformatic predictions using existing datasets from public databases. Although we employed multiple computational validation strategies (e.g., ROC analysis, single-cell validation), the identified candidate genes lack validation in our own experimental or clinical cohorts. Therefore, further studies, including in vitro and in vivo functional experiments and large-scale prospective clinical trials, are essential to confirm the diagnostic and therapeutic value of these potential biomarkers. Secondly, the 2 integrated datasets (GSE43292 and GSE100927) used different classifications of atherosclerotic lesions (Stary classification vs arterial location), which may introduce heterogeneity and potential bias into our combined analysis. While our aim was to identify common key genes across different stages and arterial beds, this inherent heterogeneity should be considered when interpreting the results.

## 5. Conclusions

In this study, a total of 5 potential diagnostic candidate genes for AS were screened and used to construct a diagnostic model, to explore the role of relevant immune genes and immune cells in AS and related mechanisms, and to provide potential therapeutic directions for the treatment of AS by building a network of atherosclerotic drugs.

## Acknowledgments

The authors sincerely thank the GEO database and all relevant researchers for sharing and publishing the data.

## Author contributions

**Conceptualization:** Ying Wei, Youdong Yang, Weiqing Sun.

**Formal analysis:** Yang Cao.

**Writing – original draft:** Yang Cao.

**Writing – review & editing:** Wenyuan Dong.

## Supplementary Material




