# Supplementary material for: Identification of immune-relevant candidate genes in atherosclerosis by WGCNA and single-cell analysis
Source: Medicine (Baltimore). 2025 Nov 14;104(46):e45871. doi: 10.1097/MD.0000000000045871 (PMC12622645; doi:10.1097/MD.0000000000045871)
Supplement: Supplementary file 1 [file medi-104-e45871-s001.docx]

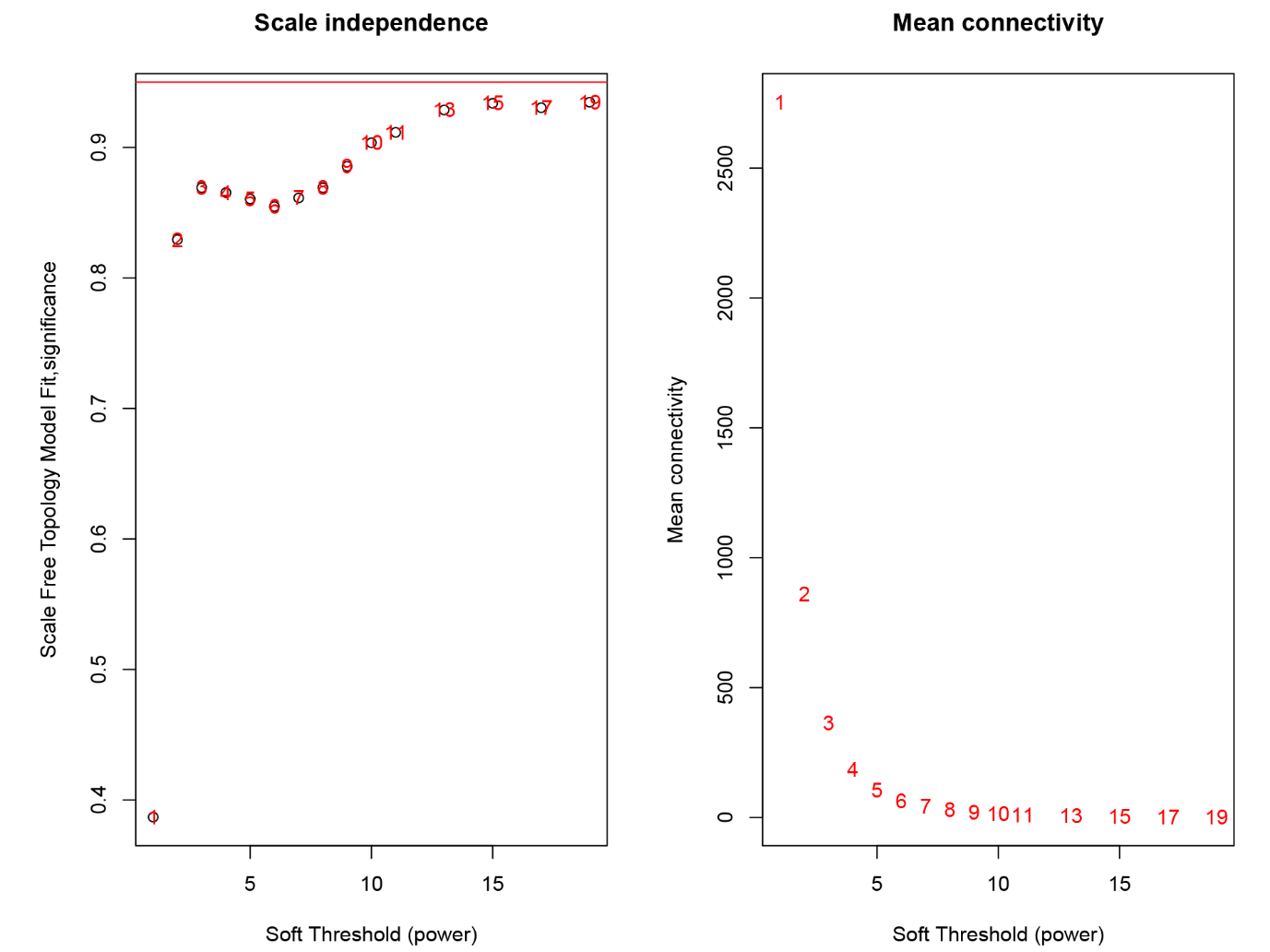


Supplementary Figure 1. Weighted Gene Co-expression Network Analysis (WGCNA) of immune inﬁltration-related genes.

Scale independence and mean connectivity across soft-thresholding powers.


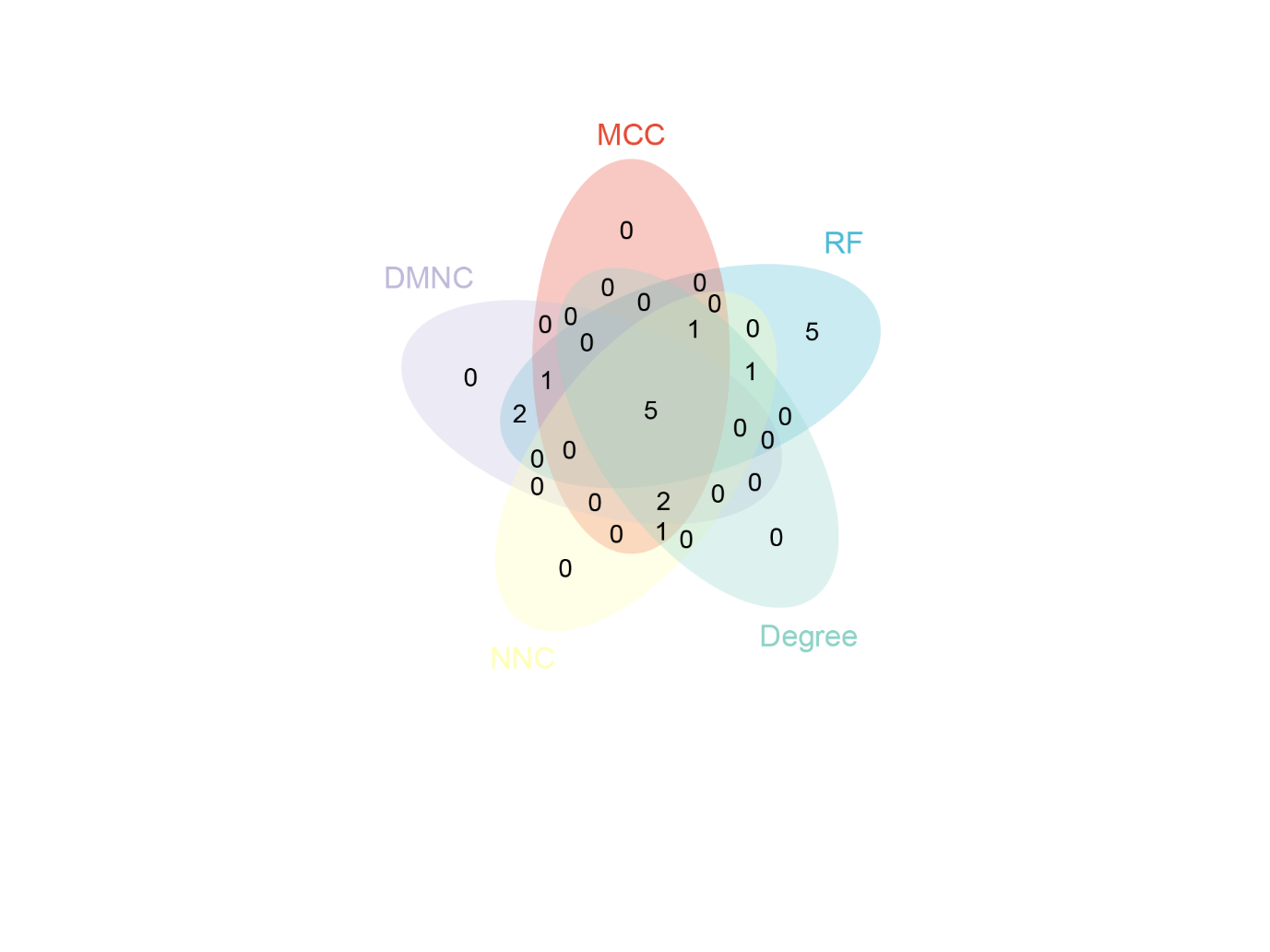


Supplementary Figure 2. Identiﬁcation of key hub genes as potential biomarkers for the disease. The analysis was

performed on a network of 17 genes using Cytoscape software.


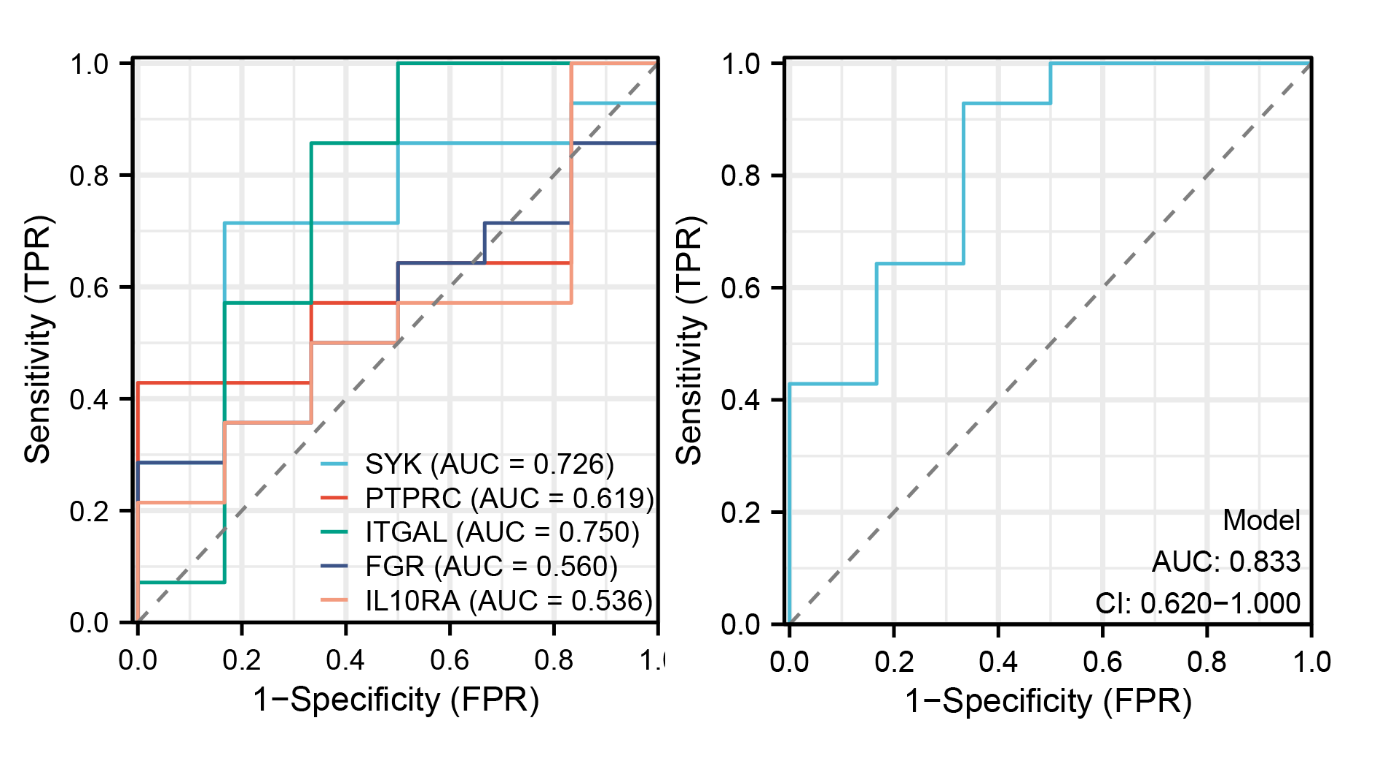


Supplementary Figure 3. Receiver Operating Characteristic (ROC) curves for individual gene markers and the combined

model. ROC curves illustrate the diagnostic performance of selected genes (SYK, PTPRC, ITGAL, FGR, IL10RA) and a

combined model in classifying samples.
